# Supplementary material for: Health facility preparedness for early detection of symptomatic cancer in Southern Africa: A multi-centre cross-sectional study
Source: PLOS Glob Public Health. 2026 May 8;6(5):e0004825. doi: 10.1371/journal.pgph.0004825 (PMC13155687; doi:10.1371/journal.pgph.0004825)
Supplement: S1 Appendix — (DOCX) [file pgph.0004825.s001.docx]

**Appendix A: Data collection tool**

**NIHR Global Research Group on Advancing Early Diagnosis of Cancer in Southern Africa: AWACAN-ED**

**HEALTH FACILITY ASSESSMENT TOOL**

**Note: Interviewer to complete this tool by reviewing relevant records and interviewing the facility manager**

**Section 1: Facility information**

- - Facility identification
  - Geographic coordinates
  - Catchment population

**Section 2: General facility infrastructure**

- - Communications, including computers, IT gadgets and internet connectivity
  - Ambulance/transport for emergencies
  - Power supply
  - Back-up electricity generator
  - Water supply
  - Waste disposal

**Section 3: Human resource capacity**

- - Number of clinical staff
  - Number of staff assessing symptomatic patients
  - Number of staff trained and available to conduct clinical breast examination
  - Number of staff trained and available to conduct pelvic examination
  - Number of staff trained and available to conduct fine needle biopsy
  - Number of staff trained and available to conduct colposcopy
  - Number of staff trained and available to conduct colonoscopy
  - Number of oncology nurses
  - Number of breast surgeons
  - Number of gynaecologic surgeons
  - Number of colorectal surgeons
  - Number of pathologists
  - Number of radiologists
  - Number of radiographers

**Section 4: Specific infrastructure and equipment**

- - Colposcopy machine
  - Mammography machine
  - Colonoscopy machine

**Section 5: Available services**

- - Oncology services
  - Pathology services
  - Radiology services

**Section 6: Medical record system**

**Section 7: Referral systems and protocols**

**Section 8: Transport to diagnostic services**

**Section 9: Feedback systems**

**Section 10: Community services**

**Section 11: Interviewer’s remarks**

**SECTION 1: FACILITY INFORMATION**

**1. Facility name:** _____________________________

**2. Province:** _____________________________

**3. Country:** ______________________________

**4. Facility GPS coordinates:**

**Latitude:** ____________ **Longitude:** _________________- **Altitude**: __________________

**5. Interviewer name:** __________________________

**6. Interview date:** ____________________________

**7. Participant number:** ___________________________

**8. Interviewee position:**

- Clinical Manager/Director
- Medical Manager/CEO
- Lead Clinician/Consultant
- HR Manager
- Other, specify__________

**9. Type of facility:**

- Tertiary hospital
- Provincial/Regional hospital
- Secondary/district hospital
- Other, specify_______

**10. Managing authority:**

- Government/public
- Private-public partnership
- Not-for-profit
- Faith-based
- Other, specify_______

**11. Facility location:**

- Urban
- Rural

**Facility catchment area and facility client load**

***Note: Field coordinator to obtain the following information and note the source of information***

|  | Catchment area information | Response | Source |
| --- | --- | --- | --- |
| a. | What is the population of the catchment area served by this facility? |  | - Hospital/facility report - Provincial health report - Other (specify) |
| b. | How many men (18 years and older) live in this catchment area? |  | - Hospital/facility report - Provincial health report - Other (specify) |
| c. | How many women (18 years and older) live in this catchment area? |  | - Hospital/facility report - Provincial health report - Other (specify) |
| d. | How many districts are served by this facility? |  | - Hospital/facility report - Provincial health report - Other (specify) |
| e. | Average number of patients/clients seen per month |  | - Hospital/facility report - Provincial health report   Other (specify) |
| f. | Number of clients with breast, cervical or colorectal symptoms seen per month (include option for not known) |  | - Hospital/facility report - Provincial health report - Other (specify) |
| g. | Total number of beds |  | - Hospital/facility report - Provincial health report - Other (specify) |
| g. | Number of oncology beds |  | - Hospital/facility report - Provincial health report   Other (specify) |
| i. | Average occupancy rate in the past month |  | - Hospital/facility report - Provincial health report   Other (specify) |
| j. | Do patients pay for any of the following (Yes/No/Not clear): |  |  |
|  | Cervical cancer screening |  | - Hospital/facility report - Provincial health report - Other (specify) |
|  | Colposcopy |  |  |
|  | Colonoscopy |  |  |
|  | Mammogram |  |  |
|  | Clinical breast examination |  |  |
|  | Laboratory service (e.g. for cytology or histology) |  |  |

**SECTION 2: GENERAL FACILITY INFRASTRUCTURE**

| **2.1 COMMUNICATIONS** |  |  |  |
| --- | --- | --- | --- |
| 1. Do all departments have a functional landline telephone? |  | - Yes - No |  |
| 2. Do all departments have a functional cellular phone? |  | - Yes - No |  |
| 4. Do all departments have a functional computer? |  | - Yes - No |  |
| 5. If yes, how many functional computers are there? |  |  |  |
| 6. Do all departments a functional email account? |  | - Yes - No |  |
| 7. Do all departments have regular internet connectivity? |  | - Yes - No |  |
| **2.2 POWER SUPPLY** |  |  |  |
| 8. Does this facility have electricity? |  | - Yes - No | |
| 9. What is the primary source of electricity? | - Power line (grid) - Generator - Solar - Other, specify___ | | |
| 10. Is a functional back‐up electricity source available? | | - Yes - No | |
| 11. If yes, what is the source of back‐up electricity? | | - Generator - Solar - Other, specify_____ | |
| 12. During the past 7 days has there been any power outage? | | - Yes - No | |
| 13. If yes, how long did the power outage last? | | - Less than an hour - 1 hour to 2 hours - >2 hours | |
| **2.3 WATER SUPPLY** |  |  |  |
| 14. Does this facility have clean running water? |  | - Yes - No |  |
| 15. What is the primary source of water? | - Piped or City Council water - Hand pump/borehole - Water tank - Other, specify______ | | |
| **2.4 AMBULANCE/TRANSPORT FOR EMERGENCIES** | | |  |
| 16. Does this facility have access to a functional ambulance service or other vehicle for emergency transport of patients? |  | - Yes - No |  |
| 17. Does this facility have access to a functional ambulance service or other vehicle for elective transport of patients? |  | - Yes - No |  |
| **2.5 EQUIPMENT** | Yes, available and functional | Yes, available but non‐ functional | No |
| 17. Does this facility have at least one refrigerator? | 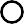  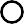 | 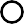  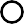 | 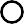  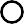 |
| 18. Does this facility have at least one autoclave or other instrument for sterilization equipment? |  |  |  |

| **2.6 SUPPLIES** | Always in stock | Out of stock in the past 30 days | Never available |
| --- | --- | --- | --- |
| 19. Soap and disinfectants | 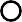 | 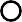 | 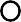 |
| 20. Paper towels and hand dryers | 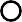 | 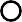 | 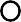 |
| 21. Alcohol hand rub | 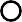 | 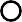 | 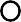 |
| 22. Disposable latex gloves | 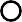 | 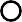 | 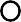 |
| 23. Face masks | 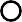 | 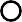 | 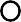 |
| 24. Gowns | 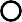 | 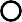 | 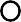 |
| 25. Goggles | 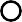 | 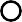 | 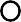 |
| 26. Has any procedure been cancelled in last 30 days due to stock out of any of these items? | | | - Yes - No - I don’t know |

| 2.6 Infection Prevention and Control (IPC) | |
| --- | --- |
| Does this facility have an IPC policy? | - Yes - No - I don’t know |
| Does this facility have a Monitoring and Evaluation (M&E) strategy for the implementation of this policy? | - Yes - No - I don’t know |
| How often are M&E activities carried out and recorded/reported at this facility | - Yes - No - I don’t know |

| **2.7 WASTE MANAGEMENT** | |
| --- | --- |
| 27. Does the facility have waste (pedal) bins with lid and plastic bin liner | - Yes - No |
| 28. Is there safe disposal of sharps? (select all applicable) | - Incineration - Open burning in protected area - Dumping without burning in protected area - Removed offsite with protected storage - No |
| 29. Is there safe disposal of biological wastes? (select all applicable) | - Incineration - Open burning in a protected area - Disposal without burning in protected area e.g., sluice room - Removed offsite with protected storage - No |

**SECTION 3: HUMAN RESOURCE CAPACITY**

| **3.1 How many of the following cadres of clinical staff are currently employed in this facility?** | Number |
| --- | --- |
| 1. Professional nurses |  |
| 1. Staff nurses |  |
| 1. Clinical nurse practitioners |  |
| 1. Oncology nurses |  |
| 1. Theatre nurses |  |
| 1. Medical officers |  |
| 1. Family physicians |  |
| 1. General surgeons |  |
| 1. Breast surgeons |  |
| 1. Gynaecologist |  |
| 1. Gynae-oncologists |  |
| 1. Colorectal surgeons |  |
| 1. Oncologists |  |
| 1. Pathologists |  |
| 1. Radiologists |  |
| 1. Diagnostic Radiographers |  |
| 1. Therapeutic Radiographers |  |
| 1. Health promotion officers/educators |  |
| 1. Total number of above cadres of staff |  |
| **3.2 How many of the following cadres of clinical staff are involved in establishing a diagnosis of breast, cervical or colorectal cancer?** |  |
| 1. Professional nurses |  |
| 1. Staff nurses |  |
| 1. Clinical nurse practitioners |  |
| 1. Oncology nurses |  |
| 1. Theatre nurses |  |
| 1. Medical officers |  |
| 1. Family physicians |  |
| 1. General surgeons |  |
| 1. Breast surgeons |  |
| 1. Gynaecologist |  |
| 1. Gynae-oncologists |  |
| 1. Colorectal surgeons |  |
| 1. Oncologists |  |
| 1. Pathologists |  |
| 1. Radiologists |  |
| 1. Diagnostic Radiographers |  |
| 1. Therapeutic Radiographers |  |
| 1. Health promotion officers/educators |  |
| **3.3 How many clinical staff in this facility have received in-service or postgraduate training in:** |  |
| 1. Assessment of patients with possible cancer symptoms |  |
| 1. Clinical breast examination |  |
| 1. Breast biopsy or fine needle aspiration (FNA) |  |
| 1. Pelvic examination |  |
| 1. Pap smear |  |
| 1. Visual Inspection with Acetic Acid (VIA) |  |
| 1. Colposcopy |  |
| 1. Colonoscopy |  |
| **3.4 How many clinical staff in this facility perform the following:** |  |
| 1. Assessment of patients with possible cancer symptoms |  |
| 1. Clinical breast examination |  |
| 1. Breast biopsy or fine needle aspiration (FNA) |  |
| 1. Pelvic examination |  |
| 1. Pap smear |  |
| 1. Visual Inspection with Acetic Acid (VIA) |  |
| 1. Colposcopy |  |
| 1. Colonoscopy |  |
| 1. When was the last time that clinical staff of this facility received training in clinical breast examination? | - This month - In the last 3 months - More than 3 months ago - Never - Don’t know |
| 1. If yes, specify which cadre of clinical staff received training in clinical breast examination (selected all that apply) | - Doctor - Nurse - Other, specify_____ |
| 1. When was the last time that clinical staff of this facility received training in pelvic examination? | - This month - In the last 3 months - More than 3 months ago - Never |
| 1. If yes, specify which cadre of clinical staff received training in pelvic examination (selected all that apply) | - Doctor - Nurse - Other, specify_____ |
| 1. When was the last time that clinical staff of this facility received training in breast biopsy or fine needle aspiration? | - This month - In the last 3 months - More than 3 months ago - Never |
| 1. If yes, specify which cadre of clinical staff received training in breast biopsy or fine needle aspiration (selected all that apply) | - Doctor - Nurse - Other, specify_____ |
| 1. When was the last time that clinical staff of this facility received training in colposcopy? | - This month - In the last 3 months - More than 3 months ago - Never |
| 1. If yes, specify which cadre of clinical staff received training in colposcopy (selected all that apply) | - Doctor - Nurse - Other, specify_____ |
| 1. When was the last time that clinical staff of this facility received training in colonoscopy? | - This month - In the last 3 months - More than 3 months ago - Never |
| 1. If yes, specify which cadre of clinical staff received training in colonoscopy (selected all that apply) | - Doctor - Nurse - Other, specify_____ |

**SECTION 4: SPECIFIC INFRASTRUCTURE AND EQUIPMENT**

| **4.1 EQUIPMENT** | Available and functional | Available but not functional | Not available | Don’t know | Not applicable | Number |
| --- | --- | --- | --- | --- | --- | --- |
| 1. Colposcopy machine |  |  |  |  |  |  |
| 1. Colonoscopy machine |  |  |  |  |  |  |
| 1. Mammography machine |  |  |  |  |  |  |
| 1. Biopsy gun |  |  |  |  |  |  |
| 1. Equipment for core biopsy (breast) |  |  |  |  |  |  |
| 1. Equipment for fine needle aspiration biopsy |  |  |  |  |  |  |
| 1. Ultrasound |  |  |  |  |  |  |
| 1. Cervical biopsy punch |  |  |  |  |  |  |
| 1. Specula |  |  |  |  |  |  |
| 1. Lithotomy beds |  |  |  |  |  |  |
| 1. Examination lamp |  |  |  |  |  |  |

**SECTION 5: AVAILABLE SERVICES**

| **5.1 Breast cancer** | |
| --- | --- |
| 1. Does this facility offer clinical assessment for possible breast cancer symptoms? | - Yes - No |
| 1. Does this facility offer breast cancer diagnostic services for women? | - Yes - No |
| 1. Does this facility offer breast cancer treatment services for women? | - Yes - No |
| 1. When is clinical breast examination conducted in this facility? | - Upon patient request/need - Daily - Once a week - Once a month - Other |
| 1. When is mammography conducted in this facility? | - Upon patient request/need - Daily - Once a week - Once a month - Other |
| 1. When is breast biopsy or fine needle aspiration conducted in this facility? | - Upon patient request/need - Daily - Once a week - Once a month - Other |
| **5.2 Cervical cancer** | |
| 1. Does this facility offer clinical assessment for possible cervical cancer symptoms? | - Yes - No |
| 1. Does this facility offer Pap smear cervical cancer screening services? | - Yes - No |
| 1. Does this facility offer Visual Inspection with Acetic Acid (VIA) cervical cancer screening? | - Yes - No |
| 1. Does this facility offer cervical cancer diagnostic services for women? | - Yes - No |
| 1. Does this facility offer cervical cancer treatment services for women? | - Yes - No |
| 1. When is pelvic examination conducted in this facility? | - Upon patient request - When a patient presents with pelvic symptoms - Daily - Once a week - Once a month - Other |
| 1. When are pap smears performed? | - Upon patient request - When a patient presents with pelvic symptoms - Daily - Once a week - Once a month - Other |
| 1. Does this facility have a colposcopy clinic? | - Yes - No |
| 1. If yes, how often is the colposcopy clinic held? | - Upon patient request - When a patient presents with cervical symptoms - Daily - Once a week - Once a month - Other |
| **5.3 Colorectal cancer** | |
| 1. Does this facility offer colorectal cancer diagnostic services? | - Yes - No |
| 1. Does this facility offer colorectal cancer treatment services? | - Yes - No |
| 1. Does this facility run a colonoscopy clinic? | - Yes - No |
| 1. If yes, how often is the colonoscopy clinic held? | - Daily - Once a week - Once a month - Other |
| **5.4 Pathology services** |  |
| 1. Do staff in this facility take biopsy or cytology specimens? **If No skip to Section 5.5** | - Yes - No |
| 1. Does this facility have a pathology lab? | - Yes - No |
| 1. If no, is there access to a lab? | - Yes - No |
| 1. If yes, how many pathologists does the lab have? | Enter number: |
| 1. How many pathology technicians does the lab have? | Enter number: |
| 1. Does the lab conduct histology or cytology? | - Yes - No |
| 1. What is the typical turn-around-time for breast histology results? | Enter duration (days/weeks/months): |
| 1. What is the typical turn-around-time for cervical histology results? | Enter duration (days/weeks/months): |
| 1. What is the typical turn-around-time for colorectal histology results? | Enter duration (days/weeks/months): |
| **5.5 Radiology services** | |
| 1. Does this facility have a radiology/radiography unit? | - Yes (if yes skip to 32) - No |
| 1. If no, Is there access to this service? | - Yes - No |
| 1. If yes, how many radiologist does the unit have? | Enter number: |
| 1. How many radiographers does the unit have? | Enter number: |
| 1. Does the unit conduct breast mammography? | - Yes - No |
| 1. Does this unit conduct breast ultrasound examinations? |  |
| 1. Does the unit conduct other cancer diagnostic imaging? | - Yes - No |
| 1. What is the typical turn-around-time for breast mammograms? | Enter duration (days/weeks/months): |
| 1. What is the typical turn-around-time for other cancer diagnostic imaging? | Enter duration (days/weeks/months: |
| **5.6 Facility tumour board** |  |
| 1. Does this facility have a tumour board? | - Yes - No (skip to section 6) |
| 1. If yes, how frequently does the board meet? | - Weekly - Bi-weekly - Monthly - Quarterly - As needed - Other, specify___________ |

**SECTION 6: MEDICAL RECORDS SYSTEM**

| 1. How are patients’ records kept in this facility? | - Paper-based - Electronic - Hybrid |
| --- | --- |
| 1. If electronic, what database system/platform is used? |  |
| 1. Is there a breast cancer register in this facility? | - Yes - No - Don’t know |
| 1. If available, how often is the breast cancer register updated? | - After each patient - Daily - Weekly - Bi-weekly - Monthly - Other ___________ |
| 1. Who enters/updates these records? | - Data entry clerk - Nurse - Doctor - Randomly assigned - Other, specify________ |
| 1. Is there a cervical cancer register in this facility? | - Yes - No - Don’t know |
| 1. If available, how often is the cervical cancer register updated? | - After each patient - Daily - Weekly - Bi-weekly - Monthly - Other |
| 1. Is there a colorectal cancer register in this facility? | - Yes - No - Don’t know |
| 1. If available, how often is the colorectal cancer register updated? | - After each patient - Daily - Weekly - Bi-weekly - Monthly - Other |
| 1. Does this facility forward cancer patients’ information/data to the district, provincial or national health departments? | - Yes, weekly - Yes, bi-weekly - Yes, monthly - Yes, whenever data is required - No - Other |
| 1. Does this facility forward cancer patients’ information/data to the district, provincial or national cancer registry? | - Yes, weekly - Yes, bi-weekly - Yes, monthly - Yes, whenever data is required - No - Other |

**SECTION 7: REFERRAL SYSTEMS AND PROTOCOLS**

| **7.1 Breast cancer** | |
| --- | --- |
| 1. Are women referred to this facility for breast cancer diagnosis? | - Yes - No |
| 1. If yes, what types of facilities are women with breast symptoms usually referred from? (select all applicable) | - Regional/district hospitals - Mission hospitals - Private hospitals - GP clinics - Primary health care centres/clinics - Other, specify __________ |
| 1. Is there a formal written protocol/guidelines for breast cancer patient referral in this facility? | - Yes - No |
| 1. Is there a formal written protocol/guidelines for breast cancer diagnosis in this facility? | - Yes - No |
| 1. What type of communication methods are used for referrals? | - Electronic referral - Telephone referral - Paper referral |
| 1. Does this facility makes referrals to other facilities for breast cancer diagnosis? | - Yes - No |
| 1. If yes, where does this facility make such referrals to? (select all applicable) | - Provincial hospital - Regional/district hospitals - Mission hospitals - Other |
| 1. Is there a breast cancer referral register in this facility? | - Yes - No |
| 1. Does this facility provide transportation for breast cancer referral? | - Yes - No |
| 1. Does this facility provide follow-up services for breast cancer referral? | - Yes - No |
| **7.2 Cervical cancer** | |
| 1. Are women referred to this facility for cervical cancer diagnosis? | - Yes - No |
| 1. If yes, what types of facilities are women with cervical cancer symptoms usually referred from? (select all applicable) | - Regional/district hospitals - Mission hospitals - Private hospitals - GP clinics - Primary health care centres/clinics - Other |
| 1. Is there a formal written protocol for cervical cancer patient referral in this facility? | - Yes - No |
| 1. Is there a formal written protocol/guidelines for cervical cancer diagnosis in this facility? | - Yes - No |
| 1. What is the origin of this guideline? | - Facility - Regional - National - International |
| 1. What type of communication methods are used for referrals? | - Electronic referral - Telephone referral - Paper referral |
| 1. Does this facility makes referrals to other facilities for cervical cancer diagnosis? | - Yes - No |
| 1. If yes, where does this facility make such referrals to? (select all applicable) | - Provincial hospital - Regional/district hospitals - Mission hospitals - Other |
| 1. Is there a cervical cancer referral register in this facility? | - Yes - No |
| 1. Does this facility provide transportation for cervical cancer referral? | - Yes - No |
| 1. Does this facility provide follow-up services for cervical cancer referral? | - Yes - No |
| **7.3 Colorectal cancer** | |
| 1. Are patients referred to this facility for colorectal cancer diagnosis? | - Yes - No |
| 1. If yes, what types of facilities are patients with colorectal symptoms usually referred from? (select all applicable) | - Regional/district hospitals - Mission hospitals - Private hospitals - GP clinics - Primary health care centres/clinics - Other |
| 1. Is there a formal written protocol/guidelines for colorectal cancer patient referral in this facility? | - Yes - No |
| 1. What is the origin of this guideline? | - Facility - Regional - National - International |
| 1. What type of communication methods are used for referrals? | - Electronic referral - Telephone referral - Paper referral |
| 1. Does this facility makes referrals to other facilities for colorectal cancer diagnosis? | - Yes - No |
| 1. If yes, where does this facility make such referrals to? (select all applicable) | - Provincial hospital - Regional/district hospitals - Mission hospitals - Other |
| 1. Is there a colorectal cancer referral register in this facility? | - Yes - No |
| 1. Does this facility provide transportation for colorectal cancer referral? | - Yes - No |
| 1. Does this facility provide follow-up services for colorectal cancer referral? | - Yes - No |

**SECTION 8: TRANSPORT TO DIAGNOSTIC SERVICES**

**Note:** This section applies ONLY to facilities where biopsy or cytology specimens are taken. Skip this section for facilities that do not collect biopsy or cytology specimens.

| 1. How are the specimens transported from this facility to the laboratory? (select all applicable) | - Facility transport - Private transport - Courier - District/ transport - Receiving lab picks up specimens - Other, specify |
| --- | --- |
| 1. Is this transport specifically for lab specimens? | - Yes - No |
| 1. When are specimens transported? | - Daily - Weekly - Monthly - Less often - As needed |
| 1. How long (on average) does it take for collected specimens to get to analysis at the referral site? | - < 1 week - 1‐2 weeks - 3‐4 weeks - >1 month |
| 1. How long (on average) does it take to receive results from the referral site? | - <1 week - 1‐2 weeks - 3‐4 weeks - >1 month |
| 1. Is there a recall system for specimens? | - Yes - No |
| 1. Does this facility receive specimens for processing from any other facilities? | - Yes - No |
| 1. How long (on average) does it take to process received specimens? | - <1 week - 1‐2 weeks - 3‐4 weeks - >1 month |
| 1. How long (on average) does it take referring facilities to receive results from this facility? | - <1 week - 1‐2 weeks - 3‐4 weeks - >1 month |
| 1. How is the communication received from the laboratory? | - Telephone - Email - Hardcopy (paper) - Internet-based (mobile app or website) - Other (specify) |

**SECTION 9: FEEDBACK SYSTEM**

| 1. Does the facility have any system of feedback from patients or community members | - Yes - No |
| --- | --- |
| 1. If yes, what form of system is it? | - Telephone - Suggestion boxes - Emails - Social media - Community forums - Other, specify__________ |

**SECTION 10: COMMUNITY SERVICES**

| **10.1 Breast cancer** | |
| --- | --- |
| 1. Does this facility provide mobile breast cancer diagnostic services? | - Yes - No |
| 1. Does this facility conduct community outreach activity to educate the community on breast cancers? | - Yes - No |
| 1. What type of community outreach or education activities do you do to promote early breast cancer diagnosis? (select all applicable) | - TV/radio - IEC materials - Posters - Social media - Community meetings - Other, specify__________ - Don't know |
| 1. Is this facility in any collaboration with a non-profit organization providing breast cancer services? | - Yes - No |
| 1. If yes, list such organizations. |  |
| **10.2 Cervical cancer** | |
| 1. Does this facility provide mobile cervical cancer diagnostic services? | - Yes - No |
| 1. Does this facility conduct community outreach activity to educate the community on cervical cancer diagnosis? | - Yes - No |
| 1. What type of community outreach or education activities do you do to promote early cervical cancer diagnosis? (select all applicable) | - TV/radio - IEC materials - Posters - Social media - Community meetings - Other, specify__________ - Don't know |
| 1. Is this facility in any collaboration with a non-profit organization providing cervical cancer services? | - Yes - No |
| 1. If yes, list such organizations. |  |
| **10.3 Colorectal cancer** | |
| 1. Does this facility provide mobile colorectal cancer diagnostic services? | - Yes - No |
| 1. Does this facility conduct community outreach activity to educate the community on colorectal cancer diagnosis? | - Yes - No |
| 1. What type of community outreach or education activities do you do to promote colorectal diagnosis? (select all applicable) | - TV/radio - IEC materials - Posters - Social media - Community meetings - Other, specify__________ - Don't know |
| 1. Is this facility in any collaboration with a non-profit organization providing colorectal cancer services? | - Yes - No |
| 1. If yes, list such organizations. |  |

**INTERVIEWER’S REMARKS**

_______________________________________________________________________________

_______________________________________________________________________________

_______________________________________________________________________________

_______________________________________________________________________________

_______________________________________________________________________________
