# Supplementary material for: Health facility preparedness for early detection of symptomatic cancer in Southern Africa: A multi-centre cross-sectional study
Source: PLOS Glob Public Health. 2026 May 8;6(5):e0004825. doi: 10.1371/journal.pgph.0004825 (PMC13155687; doi:10.1371/journal.pgph.0004825)
Supplement: S2 Appendix — (DOCX) [file pgph.0004825.s002.docx]

**Appendix B.1 Primary level Facility profile**

| **Facility** | **Population** | **Pt/month** | **#Nurses** | **Nurse:1000 patients** | **#doctors** | **Doctor:1000 patient** | **In-service training **** | **Form of record keeping** |
| --- | --- | --- | --- | --- | --- | --- | --- | --- |
| **SA Western Cape** | | | | | | | | |
| SAWCPC1 | 119889 | 5397 | 58 | 0,5 | 17 | 0,14 | Yes | Hybrid |
| SAWCPC2 | 88695 | 5000 | 55 | 0,6 | 12 | 0,14 | No | Hybrid |
| SAWCPC3 | 5000 | 2000 | 5 | 1 | 1 | 0,2 | No | Paper |
| SAWCPC4 | 16941 | 7000 | 20 | 1,18 | 4 | 0,2 | Yes | Paper |
| **Overall** | **230525** |  | **138** | **0.6** | **34** | **0.15** |  |  |
| **SA Eastern Cape** | | | | | | | | |
| SAECPC1 | 13900 | 1500 | 6 | 0,4 | 0 | N/A | No | Paper |
| SAECPC2 | 10667 | 2100 | 6 | 0,5 | 0 | N/A | No | Paper |
| SAECPC3 | 13900 | 1900 | 5 | 0,4 | 0 | N/A | No | Hybrid |
| **Overall** | **38467** |  | **17** | **0.4** | **0** | **N/A** |  |  |
| **Zimbabwe Harare** | | | | | | | | |
| ZHPC1 | 7739 | 400 | 2 | 0,3 | 0 | N/A | Yes | Paper |
| ZHPC2 | 16069 | 800 | 3 | 0,2 | 0 | N/A | Yes | Paper |
| ZHPC3 | 36968 | 6000 | 20 | 0,5 | 0 | N/A | Yes | Paper |
| ZHPC4 | 26864 | 1000 | 7 | 0,3 | 0 | N/A | Yes | Paper |
| ZHPC5 | 31001 | 300 | 5 | 0,16 | 0 | N/A | Yes | Paper |
| ZHPC6 | 80000 | 2000 | 20 | 0,25 | 0 | N/A | Yes | Paper |
| ZHPC7 | 141505 | 1400 | 18 | 0,18 | 0 | N/A | Yes | Paper |
| ZHPC8 | 116272 | 6000 | 12 | 0,1 | 0 | N/A | Yes | Paper |
| ZHPC9 | 142989 | 1080 | 45 | 0,31 | 0 | N/A | Yes | Paper |
| **Overall** | **599407** |  | **134** | **0.2** | **0** | **N/A** |  |  |
| **Zimbabwe Bulawayo** | | | | | | | | |
| ZBPC1 | 22151 | 1000 | 38 | 1,7 | 1 | 0,04 | Yes | Paper |
| ZBPC2 | 9697 | 900 | 6 | 0,6 | 0 | N/A | Yes | Paper |
| ZBPC3 | 34330 | 4500 | 29 | 0,85 | 3 | 0,09 | Yes | Paper |
| ZBPC4 | 35693 | 7000 | 42 | 1,2 | 2 | 0,05 | Yes | Paper |
| ZBPC5 | 51368 | 3500 | 42 | 0,8 | 1 | 0,02 | Yes | Hybrid |
| ZBPC6 | 63480 | 12000 | 44 | 0,7 | 1 | 0,02 | No | Hybrid |
| **Overall** | **216719** |  | **201** | **0.9** | **8** | **0.04** |  |  |

* These facilities are community-based first contact points, but also provide in-patient services: SAWC1 has 80 beds, and SAWC2 has 50 beds

** In-service training: any training related to breast, cervix or colorectal cancer

**Appendix B.2 Secondary and Tertiary Facility profile**

| **Facility** | **Population** | **Beds** | **Pt/month** | **#Nurses** | **Nurse:1000 patients** | **#doctors** | **doctor:1000 patients** | **In-service training** | **Form of record keeping** |
| --- | --- | --- | --- | --- | --- | --- | --- | --- | --- |
| **SA – Western Cape** | | | | | | | | | |
| SAWCH1 | 2151456 | 893 | * | 1675 | 0,8 | 337 | 0,16 | Yes | Hybrid |
| SAWCH2 | 700000 | 350 | 10500 | 218 | 0,3 | 51 | 0,07 | Yes | Hybrid |
| **Overall** | **2851456** |  |  | **1893** | **0.6** | **388** | **0.14** |  |  |
| **SA – Eastern Cape** | | | | | | | | | |
| SAECH1 | 3006370 | 818 | 13500 | 143 | 0,05 | 38 | 0,01 | Yes | Paper |
| SAECH2 | 1037306 | 301 | 5000 | 335 | 0,3 | 44 | 0,04 | Yes | Paper |
| **Overall** | **4043676** |  |  | **478** | **0.1** | **82** | **0.02** |  |  |
| **Zimbabwe – Harare** | | | | | | | | | |
| ZHH1 | 9338 | 152 | 2000 | 60 | 6,6 | 3 | 0,3 | Yes | Paper |
| ZHH2 | 208494 | 300 | 1200 | 210 | 1 | 6 | 0,03 | Yes | Paper |
| ZHH3 | 1849600 | 930 | 4500 | 1105 | 0,6 | 15 | 0,01 | No | Hybrid |
| ZHH4 | 540840 | 398 | 2208 | 504 | 0,9 | 82 | 0,15 | Yes | Hybrid |
| ZHH5 | 1750063 | 950 | 3500 | 1088 | 0,62 | 398 | 0,23 | No | Paper |
| **Overall** | **4358335** |  |  | **3027** | **0.7** | **482** | **0.1** |  |  |
| **Zimbabwe – Bulawayo** | | | | | | | | | |
| ZBH1 | 1539562 | 650 | 7000 | 723 | 0,47 | 172 | 0,11 | Yes | Paper |
| ZBH2 | 5707358 | 939 | 6500 | 1225 | 0,2 | 245 | 0,005 | Yes | Paper |
| ZBH3 | 250000 | 215 | 1094 | 155 | 0,62 | 7 | 0,03 | Yes | Paper |
| **Overall** | **7496920** |  |  | **2103** | **0.3** | **424** | **0.06** |  |  |
