# Supplementary material for: Health facility preparedness for early detection of symptomatic cancer in Southern Africa: A multi-centre cross-sectional study
Source: PLOS Glob Public Health. 2026 May 8;6(5):e0004825. doi: 10.1371/journal.pgph.0004825 (PMC13155687; doi:10.1371/journal.pgph.0004825)
Supplement: S3 Appendix — (DOCX) [file pgph.0004825.s003.docx]

***Appendix C.1. Availability of equipment for cancer diagnosis – Primary care (*
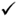
 *= available)***

| Facility | Ultrasound | Biopsy - fine needle | Vaginal Speculum | Lithotomy bed | Exam lamp |
| --- | --- | --- | --- | --- | --- |
| SA – Western Cape | | | | | |
| SAWCPC1 | **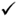** | **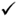** | **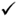** | **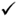** | **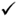** |
| SAWCPC2 |  |  | **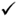** |  | **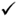** |
| SAWCPC3 |  |  | **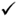** |  | **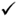** |
| SAWCPC4 | **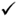** | **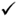** | **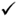** | **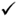** | **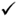** |
| SA – Eastern Cape | | | | | |
| SAECPC1 |  |  | **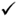** |  |  |
| SAECPC2 |  |  |  |  |  |
| SAECPC3 |  |  | **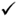** | **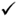** | **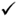** |
| Zim – Harare | | | | | |
| ZHPC1 |  |  |  |  |  |
| ZHPC2 |  |  |  | **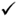** |  |
| ZHPC3 | **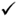** |  |  |  |  |
| ZHPC4 |  |  | **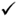** | **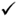** | **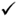** |
| ZHPC5 |  |  |  |  |  |
| ZHPC6 |  |  | **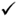** | **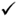** |  |
| ZHPC7 |  |  |  |  |  |
| ZHPC8 |  |  |  |  |  |
| ZHPC9 |  |  |  | **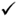** |  |
| zim – bulawayo | | | | | |
| ZBPC1 |  |  | **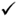** | **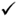** | **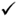** |
| ZBPC2 |  |  | **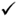** |  |  |
| ZBPC3 | **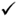** |  | **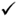** | **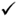** |  |
| ZBPC4 |  |  | **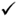** | **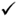** | **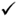** |
| ZBPC5 |  |  | **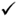** | **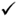** |  |
| ZBPC6 |  |  | **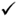** |  |  |

***Appendix C.2. Availability of equipment for cancer diagnosis – Secondary/Tertiary ( * = available)***

| Facility | Colposcope | Colonoscope | Mammo | Ultrasound | Core biopsy | Biopsy - fine needle | Punch biopsy |
| --- | --- | --- | --- | --- | --- | --- | --- |
| SA – WESTERN CAPE | | | | | | | |
| SAWCH1 | **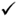** | **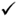** | **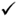** | **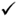** | **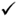** | **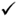** | **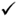** |
| SAWCH2 | **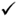** | **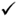** | **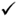** | **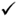** | **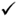** | **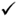** | **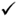** |
| SA – EASTERN CAPE | | | | | | | |
| SAECH1 | **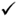** | **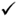** | **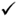** | **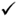** | **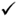** | **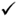** | **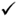** |
| SAECH2 | **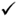** | **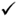** | **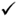** | **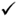** |  |  |  |
| ZIM – HARARE | | | | | | | |
| ZHH1 | **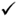** | **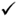** |  | **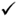** | **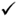** | **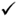** | **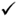** |
| ZHH2 | **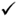** |  |  | **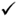** |  |  | **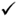** |
| ZHH3 | **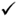** | **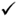** |  | **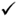** | **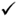** | **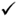** | **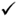** |
| ZHH4 | **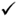** |  |  | **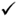** | **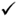** | **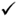** | **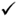** |
| ZHH5 | **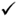** | **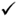** |  | **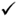** | **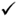** | **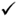** | **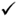** |
| ZIM – BULAWAYO | | | | | | | |
| ZBH1 |  |  |  |  | **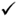** |  | **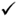** |
| ZBH2 |  | **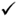** |  | **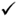** | **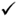** |  | **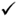** |
| ZBH3 | **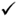** |  |  | **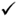** |  |  |  |
