# Supplementary material for: Health facility preparedness for early detection of symptomatic cancer in Southern Africa: A multi-centre cross-sectional study
Source: PLOS Glob Public Health. 2026 May 8;6(5):e0004825. doi: 10.1371/journal.pgph.0004825 (PMC13155687; doi:10.1371/journal.pgph.0004825)
Supplement: Attachment — Submitted filename: Response to reviewers.pdf [file pgph.0004825.s007.pdf]

12 February 2026

The editor

PLOS GPH

RE: Responses to reviewer #3

Thank you for the constructive comments received by this reviewer. We have addressed their comments as described below.

Best wishes,

Tasleem Ras – corresponding author

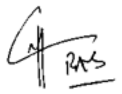

Comment	Response
In the abstract, when a term appears only once, there is no need—and no requirement—to introduce its abbreviation. The same principle applies throughout the manuscript (e.g., line 41 and line 385).	Thank you. We have reviewed the manuscript and addressed this issue. Also ensured that consistency applied throughout the study.
Please use a uniform format, spelling out numbers with numerals in parentheses for all relevant data.	Thank you for this comment. We have made the necessary amendments to the abstract
The nurse-to-1000-population ratios for primary care are inconsistent between the abstract and the Results section. These figures should be aligned.	Thank you for identifying this mistake. We have now corrected the abstract.
How are PC facilities distributed across the selected regions? It would be helpful to indicate the distances between these facilities, which would improve readers' understanding of geographical accessibility.	Thank you for this useful comment. We inserted approximate travelling times from primary to referral facility, showing the wide discrepancy between urban and rural communities. Lines 134 and 147.
It would strengthen the manuscript to include findings related to medical record systems. This topic should be incorporated into the Results and Discussion section as well, particularly in the context of future recommendations and policy implications. It would be valuable to comment on the	Thank you. We had not reported on this data, as the vast majority of facilities had reported only paper-based records. However, the following statement has now been added to the findings section (line 331): *"This was also true for the use of electronic medical records (EMR), with all facilities reporting paper-based records, except for the SA-WC facilities who indicated partial use of EMR for laboratory results, radiology reports and discharge*

current state policy in this area as well.	*summaries, while still relying on paper-based means to record daily clinical care."*<br>In discussion (Line 398): *"The persistent use of paper-based records as the main means of communication within and between facilities offers another area for possible technological intervention and efficiency gains, with subsequent system strengthening implications."*
Line 395: The reference to "Sustainable Development Goal requires clarification. Its relevance is not immediately clear, especially not reflecting in Introduction section, what are targets per country for this specific issue.	We agree with this comment. The introduction does not explore the SDG's, and reference #16 is not a primary SDG text. We have therefore modified this sentence and removed mention of the SDG's.
In the Discussion, the content in lines 378–380 would be more effective if moved to the end of the section and reframed as policy implications and recommendations. Additional recommendations should also be consolidated in this section, followed by a concluding paragraph to ensure a coherent and logical flow.	Thank you for this suggestion, which we have now implemented in full, line 442.
The title of section in line 445 should be Strength and Limitations	This has been modified in line with the reviewer's comment
