# Supplementary material for: Health facility preparedness for early detection of symptomatic cancer in Southern Africa: A multi-centre cross-sectional study
Source: PLOS Glob Public Health. 2026 May 8;6(5):e0004825. doi: 10.1371/journal.pgph.0004825 (PMC13155687; doi:10.1371/journal.pgph.0004825)
Supplement: Attachment — Submitted filename: Author responses to reviewer comments.pdf [file pgph.0004825.s006.pdf]

28 September 2025

Dear Editor,

On behalf of the authorial team, I thank you and the reviewer for the insightful comments received. I have responded in detail to each comment/recommendation, and outline those below.

I hope that these meet with your favourable consideration.

Best wishes,

A/Prof Tasleem Ras

University of Cape Town, South Africa

Editor comments:

1. Please include a complete copy of PLOS' questionnaire on inclusivity in global research in your revised manuscript.

   This has now been included

2. Please amend your detailed Financial Disclosure statement.

   Statement has been amended to include all author contributions. Line 469-477

3. Please send a completed 'Competing Interests' statement

   Statement has been uploaded separately and included in the manuscript in line 481-482

4. All PLOS journals now require all data underlying the findings described in their manuscript to be freely available to other researchers, either
   1. In a public repository,

2. Within the manuscript itself, or
3. Uploaded as supplementary information – *the dataset has been uploaded as a supplementary file*

5. Please provide separate figure files in .tif or .eps format.

   Figures uploaded in .tif format

6. We have noticed that you have uploaded Supporting Information files, but you have not included a list of legends. Please add a full list of legends for your Supporting Information files after the references list.

   List of legends have been added after the reference list in the manuscript from line 637 onwards

---

Reviewer #1 comments:

The manuscript addresses an important gap by assessing health facility preparedness for early cancer detection in Southern Africa. The study is novel for the region and uses a comprehensive health facility assessment tool aligned with WHO's framework. The findings are relevant for policymakers and health systems planners. The manuscript is generally well-written and structured, but there are areas that would benefit from clarification, methodological strengthening, and deeper discussion.

1. Given that facility selection was purposive for primary care and exhaustive for secondary/tertiary facilities within the chosen referral pathways, how representative are the findings for the broader regions or for each country as a whole?

This issue is dealt with in line 434-436 in the "limitations' section: "The non-random sampling, and small sample size within the geographic areas studied limits our ability to claim that these findings are generalisable to all facilities in SA, Zimbabwe or sub-Saharan Africa."

2. How were terms like "adequate communication infrastructure" and "community engagement" operationally defined? Was there a scoring threshold, and if so, how was it determined?

Thank you. We did not initially define a scoring threshold to determine 'adequacy' of communication infrastructure. We considered this comment and decided that the

presence of 3/5 items within this construct would be deemed adequate. This is now reflected in the abstract in line 52-53.

Community engagement has now been defined in line 339-341.

3. Given the substantial gap in colorectal cancer readiness at primary care level (only 9% providing clinical assessment), how do the authors recommend prioritizing this against breast and cervical cancer services which already have some infrastructure?

Thank you. We agree with the comment, and addressed this in line 398-406: "This represents an area that requires significant attention, capacity-building and resource allocation, given the high prevalence 8-9 and mortality rates associated with advanced colorectal cancer at diagnosis in Africa.10-13 The use of targeted faecal immunohistochemical tests (FIT) as a non-invasive accurate test, may assist remote PC facilities in identifying symptomatic patients needing rapid escalation of care.32-34 In the context of low-resourced PC, introducing FIT would need careful exploration from the perspectives of the health system, clinical application and economic feasibility."

4. Minor style issue: Define ST (secondary/tertiary) in the abstract for readers unfamiliar with the term.

Thank you. This has been done: line 42

5. The recommendations include mHealth innovations, but implementation feasibility in low-connectivity areas is challenging. Did the authors assess any existing successful pilots in similar contexts that could be referenced?

Thank you for this comment. However, we have already included this in the discussion section – we cite studies that explored feasibility in African contexts: line 387-394 "The use of mobile technologies not dependent on fixed line infrastructure may be a useful short-term intervention, though early evidence from various African contexts indicates significant challenges that still need to be addressed in this arena.[29-31] *The challenges identified by these studies included limited mobile coverage, low technological skills amongst healthcare workers, and inconsistent power supply to remote areas. Designing context-specific mobile applications, training healthcare workers in the integration of communication devices into their clinical duties and ensuring a steady power supply were some pragmatic and feasible solutions proposed to bridge the infrastructural gap."*
